# Stability and change in genetic and environmental influences on depressive symptoms in response to COVID-19 pandemic

**DOI:** 10.3389/fpsyt.2023.954737

**Published:** 2023-02-13

**Authors:** Angelo Picardi, Corrado Fagnani, Antonella Gigantesco, Virgilia Toccaceli, Maria Antonietta Stazi, Emanuela Medda

**Affiliations:** Centre for Behavioural Sciences and Mental Health, Italian National Institute of Health, Rome, Italy

**Keywords:** depression, mental health, COVID-19, twin, longitudinal survey, genetic and environmental factors

## Abstract

**Background:**

The rapid spread of the new Coronavirus and the consequent restrictions to contain transmission generated an unprecedented psychological impact on the general population. The Italian Twin Registry performed a longitudinal study to investigate to what extent genetic and environmental influences contributed to changes in depressive symptoms.

**Methods:**

Data from adult twins were collected. All participants completed an online questionnaire including the 2-item Patient Health Questionnaire (PHQ-2) just before (February 2020) and immediately after the Italian lockdown (June 2020). Genetic modeling based on Cholesky decomposition was used to estimate the role of genetic (A) and both shared (C) and unshared (E) environmental factors in the observed longitudinal course of depressive symptoms.

**Results:**

Longitudinal genetic analysis was based on 348 twin pairs (215 monozygotic and 133 dizygotic pairs) with a mean age of 42.6 years (range 18–93 years). An AE Cholesky model provided heritability estimates for depressive symptoms of 0.24 and 0.35 before and after the lockdown period, respectively. Under the same model, the observed longitudinal trait correlation (0.44) was approximately equally contributed by genetic (46%) and unshared environmental (54%) influences, while longitudinal environmental correlation was lower than genetic correlation (0.34 and 0.71, respectively).

**Conclusions:**

Although the heritability of depressive symptoms was rather stable across the targeted time window, different environmental as well as genetic factors seemed to act before and after the lockdown, which suggests possible gene-environment interaction.

## Introduction

The World Health Organization declared the outbreak of a pandemic on March 2020 (1). COVID-19 was a novel disease with an incompletely described clinical course and without proven effective specific treatment strategies. Healthcare professionals were exposed to this highly contagious virus and heavily burdened by the excessive workload. People with respiratory symptoms were encouraged to isolate themselves and social distancing was recommended to all population. The SARS-CoV2 spread had also a huge economic impact. Commercial activities stopped almost entirely during lockdown, and many people lost their jobs or faced a decrease in their income (2).

All these direct and indirect effects of the COVID-19 pandemic had an unprecedented repercussion in almost every country. Preliminary findings from research performed in China, where the outbreak originated, soon highlighted a huge psychological impact of the pandemic on individuals (3, 4). Further general population studies yielded similar findings in many other countries. In a Spanish longitudinal study, participants showed increased levels of anxiety, depression, and stress throughout the confinement imposed to counter the virus spread (5). Similarly, an observational population-based study conducted in the UK in 2017–2019 and in 2020, suggested a change in the prevalence of mental health problems attributable to the COVID-19 crisis (6). These and other studies also highlighted that the mental health impact was greater among younger people, women, people living alone, and people with poor social support or low income (7–9).

Italy was one of the first countries to be hit by the pandemic, and the Government promptly introduced strict measures to contain the outbreak. The beginning of a nationwide lockdown was declared in March 2020 and its end in May 2020. A number of studies suggest that the experience of lockdown led to the development of several mental health problems, particularly depressive symptoms (9–11).

Although many studies examined the mental health impact of COVID-19, to our knowledge only few studies investigated the longitudinal course of psychological and behavioral traits and possible pandemic-related gene-environment interactions. The twin study method is indeed a proper approach to disentangle the genetic and the environmental components of considered traits and thus to estimate their heritability. In a longitudinal setting, the twin study method also allows to make inferences on the stability, change or interactions of these components over time.

The traits of optimism and meaning in life were actually investigated in a Dutch twin study, which detected a lower heritability during the first months of the pandemic compared to the pre-pandemic period, and also suggested possible gene-environment interaction (12). Another study (13) focused on a broad range of psychological measures collected in 2018 and after the lockdown. That study reported largely stable individual differences from pre- to post-lockdown and suggested that genetic influences, which accounted for more than half of the phenotypic stability, were the major systematic force shaping individual differences in psychological traits at pre- as well as at post-lockdown.

This study aimed at exploring possible changes in the gene-environment architecture of depressive symptoms in a cohort of Italian twins surveyed immediately before and after the lockdown.

## Methods

### Participants and procedures

The study sample was derived from the population-based Italian Twin Register (ITR) (14), which currently includes information on more than 29,000 twins belonging to different age groups and geographical areas, who gave their written consent to be contacted for participation in ITR research projects. The sample includes 1,690 twins who participated in two surveys: (i) a chronic pain survey, just before the beginning of the lockdown, when risk perception among the general population was still very low (February 2020); (ii) a COVID-19 survey, immediately after the end of this lockdown (June 2020), aimed at investigating the physical and mental health impact of the pandemic on Italian adults (9).

The study sample comprised 696 twins from 348 complete pairs [215 monozygotic (MZ) and 133 dizygotic (DZ) twin pairs]. Participants' mean age was 42.6 ± 14.5 years, and 70% of them were female.

### Measures

Depressive symptoms were assessed at both time points with the 2-item Patient Health Questionnaire (PHQ-2), which is a validated depression screener in the general population (15). The PHQ-2 includes the first two items of the PHQ-9 and measures the degree to which an individual has experienced depressed mood and anhedonia over the past 2 weeks. In settings such as primary care or some inpatient and outpatient specialty care, the PHQ-2 has been widely validated and found to be up to 87% sensitive and 77% specific using a threshold score of 2 or greater and up to 62% sensitive and 92% specific using a threshold score of 3 or greater, in studies that used fully structured interviews as reference standards (16). In studies that used semi-structured interviews, PHQ-2 sensitivity and specificity were 0.72 and 0.85 for cut-off scores of 3 or greater (16). A recent individual participant data meta-analysis of 44 studies involving 10,627 participants reported only small differences in sensitivity and specificity between the PHQ-2 and the PHQ-9, which suggests that the psychometric performance of the PHQ-2 is only marginally lower than that of the full PHQ-9 (16).

### Statistical analysis

First, we performed a statistical analysis based on twins as individuals. Prevalence rates of depressive symptoms (based on PHQ-2 scores ≥ 2) observed at wave 0 and wave 1 were compared by the McNemar's test. Moreover, a linear regression model was fitted to predict PHQ-2 scores based on age and sex of participants, at first- and second-wave separately. Beta coefficients (ß), 95% confidence intervals (95% CI) and *P*-values for the relation between depression scores and age or sex were estimated taking into account the clustered (i.e., paired) structure of the data.

At both time points, the PHQ-2 score was calculated for each twin and was used in subsequent twin analysis, which was restricted to complete pairs only. This analysis is based on the fact that MZ twins share identical genes, whereas DZ twins share, on average, 50% of their segregating genes; in this scenario of genetic relatedness, a higher within-pair trait correlation in MZ compared to DZ pairs indicates a genetic influence on the trait. Statistical significance for the difference in correlation by zygosity group was tested based on Z test after Fisher's r-to-Z transformation. In correlation analysis, the (within-individual/cross-wave) correlation between the two waves was estimated as a measure of trait longitudinal stability; moreover, the (cross-twin/within-wave) correlation between twins and their cotwins at each wave and the (cross-twin/cross-wave) correlation between twins at a given wave and their cotwins at the other wave were estimated in MZ and DZ pairs separately, in order to gain insights into the genetic role in trait expression at each wave and in trait longitudinal stability.

Bivariate Cholesky decomposition models including additive genetic (A), shared environmental (C) or non-additive genetic (D), and unshared environmental (E) components were fitted to the longitudinal data. The A component represents the additive effects of all alleles that influence the trait, without interactive effects; the D component represents interactions between alleles at the same locus (dominance) or at different loci (epistasis); C represents the effects of environmental factors that are shared by the twins within the family—particularly during childhood/ adolescence (e.g., rearing environment, parental behaviors, etc.)—or in the womb (e.g., hormonal exposures); E represents the effects of environmental factors that are unique to an individual (e.g., lifestyles, infections, etc.), including measurement error (17).

The Cholesky model postulates the presence of common genetic and/or environmental contributions that create the correlation between the waves. For two waves, a Cholesky decomposition includes two independent genetic and environmental factors: the first factor loads on both waves, the second factor loads only on the second wave. This model provides the fullest explanation of the data because it does not impose any restrictions on the genetic and environmental contributions to covariation. By this model, it is possible to estimate: (i) The proportion of total trait variance at each wave that is explained by genetic variance (i.e., the “heritability”) and environmental variance; these proportions indicate how much of the observed individual differences at a given wave is due to genetic and environmental differences. (ii) The proportions of cross-wave correlation that are attributable to common underlying genetic factors (i.e., the “longitudinal heritability”) and to common environmental factors; these proportions indicate how much of the observed stability is due to genetic and environmental stability. (iii) How many of the same genes (i.e., the “genetic correlation”) or of the same environmental factors (i.e., the “environmental correlation”) are involved in trait expression at each wave; values significantly lower than 1 indicate that genetic or environmental “innovations” come into play at the second wave compared to the first one.

Saturated and bivariate genetic models included age and sex as covariates, and were fitted by the classical version of the Mx software (18). Genetic and environmental parameters' estimates were derived from the best-fitting model, which was selected based on the Akaike Information Criterion.

## Results

Mean PHQ-2 scores were significantly (*P* < 0.01) higher immediately after the lockdown (mean: 1.40, Standard Deviation—SD: 1.37; June 2020) than before lockdown (mean: 1.20, SD: 1.33; February 2020), and the prevalence of depressive symptoms increased from 36.6 to 43.1% (*P* = 0.004).

Linear regression analysis showed a significant negative relationship between PHQ-2 scores and age. In more detail, higher levels of depression in younger subjects were observed. Moreover, the effects of age on depression scores are similar in magnitude before and after lockdown (wave 0: ß = −0.018, 95% CI −0.025; −0.011, *P* < 0.001, *N* = 696; wave 1: ß = −0.019, 95% CI −0.027; −0.012, *P* < 0.001, *N* = 696).

Sex did not reach a statistically significant association with PHQ-2 scores at either wave.

Table 1 shows the results of correlation analysis and genetic modeling.

**Table 1:** Correlation patterns and best-fitting model's estimates for depression symptoms.

**Correlations**			
**Cross-Twin/within-wave**			
**Wave** **0**		**Wave** **1**	
**MZ**	**DZ**	**MZ**	**DZ**
0.25 (0.13; 0.37)	0.04 (−0.13; 0.21)	0.35 (0.23; 0.46)	0.13 (−0.04; 0.29)
**Within-Twin/cross-wave**			
	0.44 (0.38; 0.50)	
**Cross-Twin/cross-wave**			
	**MZ**	**DZ**	
	0.23 (0.13; 0.32)	0.09 (−0.12; 0.14)	
**Best-fitting (AE) model estimates**			
**Proportions of variance**			
**Wave** **0**		**Wave** **1**	
**A**	**E**	**A**	**E**
0.24 (0.12; 0.35)	0.76 (0.65; 0.88)	0.35 (0.23; 0.45)	0.65 (0.55; 0.77)
	**Proportions of covariance**		
	**A**	**E**	
	0.46 (0.26; 0.64)	0.54 (0.36; 0.74)	
	**Correlations**		
	**r**_a_	**r**_e_	
	0.71 (0.47; 0.96)	0.34 (0.23; 0.44)	

Values given in parentheses indicate 95% confidence intervals.

A, additive genetic variance/covariance; E, unshared environmental variance/covariance; Genetic correlation r_a_; Environmental correlation r_e_.

Cross-Twin/Within-Wave correlation of PHQ-2 scores was significantly higher in MZ compared to DZ twins, both at wave 0 (*P* = 0.03) and at wave 1 (*P* = 0.02). Cross-wave correlation was estimated to be 0.44, which suggests only a moderate stability of depressive symptoms across the lockdown. MZ cross-twin/within-wave correlations were substantially greater than the corresponding DZ correlations, which indicates genetic effects at both time points. A higher cross-twin/cross-wave correlation in MZ compared to DZ pairs was consistent with a genetic contribution to the observed longitudinal stability.

A bivariate Cholesky model including only additive genetic (A) and unshared environmental (E) components best fitted the data. The contribution of shared (familial) environment to model fit was negligible, as also indicated by the modest proportions of variance provided at each wave by the full (ACE) model (2% at wave 0, 5% at wave 1). Under the best-fitting (AE) model: (i) the heritability estimate was rather stable over time (0.24, wave 0; 0.35, wave 1); (ii) genetic and environmental factors accounted for 46 and 54% of the longitudinal stability, respectively; (iii) environmental and genetic correlations between waves were both significantly less than perfect, with the longitudinal environmental correlation being much weaker than the genetic one (0.34 and 0.71, respectively).

All the results on the correlational pattern and the underlying gene-environment structure remained basically unchanged when including also unmatched twins in the analyses.

## Discussion

Consistently with previous research, we observed an increase in the severity of depressive symptoms in the initial phase of the pandemic. Most studies from the USA and Europe reported a small to moderate deterioration in population mental health during the early phases of the pandemic, with a more severe worsening of mental health in particular groups (5–7, 9).

The longitudinal bivariate Cholesky decomposition analysis showed that additive genetic influences moderately explain the variance in depressive symptoms both before and during the pandemic, with the remaining substantial proportion of variance explained by the non-shared environmental component at each time point. Shared environment seemed to play a negligible role both in time-specific individual differences and in the observed longitudinal pattern, as it is usually the case with samples of adult age where familial effects tend to vanish and to be superseded by accumulating individual-specific experiences. Both genetic and environmental effects were moderately comparable over time. Specifically, during the lockdown the effect of heritability was higher and the effect of environmental factors was lower than before the pandemic. This could be due to qualitative gene-environment interaction, meaning that different genes may have been involved before and during the lockdown.

The sizes of the estimates of genetic and environmental effects were relatively consistent with the estimates reported in previous studies using a variety of mental health instruments including measures of anxiety and depressive symptoms (13) and measures of wellbeing (12). In a longitudinal twin study examining mental health in twins in their mid-twenties, prior to the pandemic (2018) and during the pandemic (April, July, October 2020, and March 2021), the authors reported slightly higher heritability estimates than ours, specifically 33% before the pandemic and 40% in April 2020, 1 month after the first British lockdown (13). Consistently with our findings, a Dutch adult twin study (12) reported quite comparable heritability estimates for optimism and meaning in life before the pandemic (26 and 32%, respectively) and in April–May 2020, (20 and 25%, respectively). However, at variance with our results, that study reported relatively lower heritability and higher relative effect of environmental factors during the pandemic than before.

In our longitudinal analyses, the phenotypic cross-wave correlation was 0.44, which suggests that symptom liability was only moderately stable across the lockdown period. In the Dutch study, the longitudinal stability was even lower, as the correlation between time points was 0.36 for both optimism and meaning in life. In contrast, in the British study a higher phenotypic correlation for several psychological measures was observed, though for depression the longitudinal correlation was only slightly higher (0.55) than that we observed (13).

In our study, the relative contributions of genetic and environmental factors to the covariance in depressive symptoms between waves (46 and 54%, respectively) were moderate, which suggests that both environmental influences and, to a lesser extent, genetic influences were moderately contributing to stability.

The relatively high cross-wave genetic correlation (0.71) indicates a substantial overlap for additive effects at the two time points and suggests that many genetic factors contributing to individual differences endured over time. This result also provides an indication for possible qualitative gene-environment interaction effects. One may hypothesize that the experience of strict pandemic measures may have changed, although only moderately, a person's genetic risk of depressive symptoms, given that different genes may have expressed their influence in response to the pandemic. It should be noted that part of the genetic stability we found in our study may have been accounted for by stable heritable characteristics correlated with susceptibility to depression, such as some personality traits. However, we could not directly test this hypothesis because we had no information on these characteristics.

In contrast, the low cross-wave environmental correlation (0.34) indicates that the overlap of the environmental influences between the two time points was small, and that environmental factors were mostly unique to each time point, as could be expected given the changing environmental impact of an event such as a pandemic. One may hypothesize that new individual-specific experiences related to the pandemic may have played a key role in the observed increase in depressive symptoms levels.

It should be acknowledged that the use of a 2-item instrument to measure the severity of depressive symptoms implies some reduction in psychometric reliability, given the known correlation between reliability and number of items. However, this does not appear to be a major limitation, since a number of studies have reported acceptable to good test-retest reliability for the PHQ-2, ranging from 0.70 to 0.86, with an interval from the initial administration ranging from 1 to 4 weeks later (19–23).

In previous studies, it was also observed that PHQ-2 scores did not substantially change even over a longer period of time, which suggests that it is a relatively stable measure, even though it assesses a state rather than a trait variable. Specifically, two studies, in which the PHQ-2 was administered to a number of primary care patients with epilepsy, found that depression scores remained stable over a mean interval of 4 months (24) and even more (25).

A more recent study has compared the screening yield of both the PHQ-2 and the PHQ-9 by means a retrospective analysis of the PHQ-9 data monthly collected over a period of nine consecutive months (from June 2018 to February 2019) in patients attending a pediatric clinic who were aged 12–21 years. That study found that the yield of both questionnaires remained stable over the first 3 months of the testing period. Of note, after the first 3 months, the PHQ-9 average scores increased while average PHQ-2 scores continued to be stable (26). Partially in contrast with the above-mentioned finding regarding the PHQ-9, in a recent longitudinal study involving a European-wide sample of patients after Traumatic Brain Injury (TBI), the analysis showed high stability of depression scores as measured by the PHQ-9 at 3-, 6-, and 12-months post-TBI assessments (27). Accordingly, another longitudinal study demonstrated high retest stability of the PHQ-9 over a 1-year period among adults with physical disabilities (28).

Other limitations of this study are the exclusive reliance on a self-report measure, the relatively small sample size, and the availability of data from only two time points.

While these limitations suggest some caution in interpreting our results, this study suggested the salience of unique idiosyncratic experiences and, to a lesser extent, novel heritable factors, which during the early phases of the COVID-19 pandemic may have come into play in affecting changes in depressive symptoms. It is important to point out that longitudinal twin analysis based on latent-variable models (which is the approach used in this study) does not directly allow to identify these specific modifiable environmental factors; however, the results of such studies may provide relevant information for research in this direction and for the feasibility of prevention strategies.

The effects of the pandemic might further change over time. The individual's liability to depressive symptoms may become more stable if relatively steady genetic factors were to play a larger role in the subsequent phases of the pandemic. To shed more light on this topic, future longitudinal twin studies should examine longer periods and collect data over three or more time points.

## Data availability statement

The original contributions presented in the study are included in the article/supplementary material, further inquiries can be directed to the corresponding author.

## Ethics statement

The studies involving human participants were reviewed and approved by Ethical Committee of the Istituto Superiore di Sanità. The patients/participants provided their written informed consent to participate in this study.

## Author contributions

AP, CF, AG, MS, and EM contributed to conception and design of the study. EM organized the database. EM and CF performed the statistical analysis. AP, CF, AG, VT, and MS wrote sections of the manuscript. All authors contributed to manuscript revision, read, and approved the submitted version.
